# Influence of the casing layer on the specific volatile compounds and microorganisms by *Agaricus bisporus*

**DOI:** 10.3389/fmicb.2023.1154903

**Published:** 2023-05-17

**Authors:** Yong-Hui Wang, Xiao-Ying Yang, Lu-Zhang Wan, Hai-Xia Ren, Ling Qu, Hui-Dong Guo, Li-Li Dong, Xiao Lu, Peng-Fei Ren

**Affiliations:** ^1^Key Laboratory of Wastes Matrix Utilization, Ministry of Agriculture, Institute of Agricultural Resource and Environment, Shandong Academy of Agricultural Sciences, Jinan, China; ^2^College of Food Science and Technology, Qilu University of Technology (Shandong Academy of Sciences), Jinan, China

**Keywords:** casing layer, *Agaricus bisporus*, yield, volatile compound, microorganisms

## Abstract

One of the major variables affecting yield of the mushroom *Agaricus bisporus* is the casing layer, which directly affects the productivity and mass. Here, volatile organic compounds were extracted by headspace solid-phase microextraction and high-throughput sequencing was used to analyze the microbial community diversity. The relationship between mushroom yield at different cropping stages and the contents of volatile organic compounds and microorganisms in three different casing layers: peat, peat + soil and soil were systematically evaluated. The result shows that Benzaldehyde and (E)-2-octenal which stimulate yield, obviously increased as mushrooms grew, while 3-octanone, which inhibits yield, decreased over time in all three casing layers. However, there was not a strong correlation between the concentration of volatile compounds and yield. In addition, more than 3,000 bacterial operational taxonomic units (OTUs) by performing high throughput sequencing of the microbes were obtained in the three casing layers. Interestingly, the microbial community compositions were very similar between the three casing layers at a later cropping stage, but the community richness varied significantly in different casing layers and at different cropping stages. At the phylum level, the communities had similar structures but were quantitively very different, and this was even more obvious at the genus level. Principal component analysis revealed significant alterations in microbial community structure in different casing layers. *Sphingomonas*, *Dongia* and *Achromobacter* were the dominant genera at cropping stage 1, and the stage 3 were abundant in *Saccharibacteria_norank, Pseudomonas*, *Flavobacterium* and *Brevundimonas*, which was positively correlated with yield, while the abundance of *Pseudomonas* at stage 1 and *Lactococcus* and *Bacillus* at stage 3 was negatively correlated with yield. These results provide a guide for the development and agricultural application of microbial agents for yield improvement in the production of *A. bisporus*.

## 1. Introduction

*Agaricus bisporus*, or button mushroom, is an edible mushroom species cultivated worldwide (Toker et al., 2007). It has been difficult to meet the growing demand for *A. bisporus* even though there has been about a 25-fold increase in yield in recent years (Royse, 2014). Researchers have tried many techniques to improve yield, such as strain selection (Tschierpe, 1983), irrigation with calcium chloride (Diamantopoulou and Philippoussis, 2001) and optimization of agronomic techniques (Royse and Chalupa, 2009). Since a casing layer with specific physical, chemical and microbiological properties is required to stimulate *A. bisporus* primordium initiation and sporophore formation (Eger, 1961; Flegg and Wood, 1985), many studies have focused on manipulating casing materials to improve yield (Gülser and Pekşen, 2003; Colak, 2004; Yang et al., 2019; Carrasco and Preston, 2020).

The casing layer is a major factor affecting yield, quality and uniformity of commercial cropping (Noble and Gaze, 1995). Researchers have attempted to identify the factors in the casing layer that can impact button mushroom production, such as the volatile compounds released by the mushroom, the osmotic and matric potentials, the water holding capacity, the pore space, the substrate content and the types of microorganisms in the casing layer (Noble et al., 1999; Beyer et al., 2002; Pardo et al., 2010). The major microbial stimulus affecting sporophore formation in the casing layer is *Pseudomonas putia* (Hayes et al., 1969), which is necessary for primordium initiation in the model strain *Agaricus bisporus* W19 (Rainey et al., 1990). Inoculation of axenic casing materials with different *Pseudomonas* isolates also induces primordium initiation, but the number of primordia is significantly lower than that in non-axenic casing containing specific microorganisms (Noble et al., 2009), which suggests that some factors other than the microbial stimulus are also important for primordium initiation.

The interaction between bacteria and fungi during mushroom cultivation has been described, which depend on the bacterial isolate and the developmental stage of the fungus (Carrasco and Preston, 2020). Wood and Blight, M. (1983) hypothesized that some casing layers can absorb compounds that inhibit primordial initiation, and the use of these casing layers may potentially obviate the requirement for stimulatory *Pseudomonas* (Long and Jacobs, 1974). For example, 1-octen-3-ol, a volatile C8 compound characteristic of mushrooms that can inhibit primordium initiation, is easily absorbed by activated charcoal (Grove, 1981; Wood and Blight, M., 1983). Consistent with this, researchers found that *Agaricus bisporus* can produce primordia and mature sporophores on an axenic casing supplemented with charcoal (Noble et al., 2003). Moreover, the pseudomonad populations in the casing layer metabolize volatile C8 compounds that inhibit primordium initiation, such as 1-octen-3-ol and 2-ethyl-1-hexanol released by mycelia or the substrate, enabling primordium initiation. Therefore, the stimulatory effect of the casing layer is partly due to the removal of inhibitory C8 compounds by pseudomonad populations or absorption of these compounds by the casing layer (Noble et al., 2009). Chen et al. (2013) analyzed the synergism between bacteria and fungi, results showed that 1-aminocyclopropane-1-carboxylic acid (ACC) deaminase (AcdS)-producing bacteria were abundant in the casing soil of *A. bisporus*. Zhang et al. (2019) introduced a bacterial *Pseudomonas* sp. UW4 *acdS* gene into *A. bisporus*, and the heterogeneously expressed bacterial *acdS* gene reduces ethylene-synthesis, eliminating ethylene inhibition on the mycelium growth and primordium formation in *A. bisporus*. However, the relationship between volatile compounds, microorganisms (e.g., pseudomonads) and the final yield still remains unclear. Other bacteria such as *Arthrobacter terregens*, *Bacillus megaterium* and *Rhizobium meliloti* and their metabolites can also trigger sporophore formation (Park and Agnihotri, 1969). Photosynthetic bacteria such as *Rhodopseudomanas palustris* can increase mushroom yield while *Alcaligenes* sp. can stimulate primordium formation (Han, 1999; Fermor et al., 2000).

The study has found that bacterial PLFA levels are higher at the primordia formation stage and that some gram-negative bacteria may play important roles in sporophore initiation, which phosphorlipid fatty acid (PLFA) analysis is a reliable tool to identify the microbial community structure of the casing soil during *Agaricus bisporus* cropping (Cai et al., 2009). However, the identity of these microorganisms is still poorly documented. In this study, the relationship between mushroom yield at different cropping stages and the contents of volatile organic compounds (VOC) and microorganisms in three different casing layers were evaluated. Our aim is to discover specific volatile compounds and microorganisms that affect the final yield in three kinds of casing layers: peat, peat + soil or soil. We found that these casing layers result indifferent mushroom yields, which are correlated with specific volatile compounds and microorganisms in the casing layer.

## 2. Materials and methods

### 2.1. Casing layers and mushroom culture

*Agaricus bisporus* strain W192, maintained by the Institute of Agricultural Resources and Environment, Shandong Academy of Agriculture Sciences, was chosen for this study because it is a major commercial cultivar grown in China. The experiment was carried out at FuBang Mushroom Industrial Park in Shenxian, Shandong Province. Compost was made from wheat straw (65%), rice husks (10%), chicken manure (22%), lime (2%) and gypsum (1%); all proportions are w/w. Phase I and Phase II of the composting process were conducted in a tunnel for 10 and 8 days, respectively. Compost (20 kg) packed in polyethylene bags was inoculated with 0.175 kg spawn, and then transferred to a growing room. For spawn run, the air temperature was maintained at 22 ± 2°C, the air relative humidity was maintained at 70% and the air CO_2_ concentration was 1,500 ppm. Three types of casing materials, peat, peat + soil (1:2, v/v) and soil were added to the surface of fully colonized Phase II compost to a height of 2 cm after spawn run. Then the air temperature was adjusted to 15 ± 2°C, the air relative humidity was adjusted to 85%, and the air CO_2_ concentration was decreased to 800 ppm for mushroom production. The casing layers were then collected at stage 1 and stage 3 of sporophore development (Hammond and Nichols, 1975) with three replicates for volatile organic compound and metagenomics analysis. At stage 1, the diameter of the pileus was ≤5 mm, like “pinhead,” with the velum not differentiated. At stage 3, the diameter of the pileus was approximately 30–40 mm, resembling a “closed cup,” with the velum stretched but still intact. Yields per unit of compost (kg of first flush and second flush mushrooms harvested from 1 m^2^ of compost) were recorded.

### 2.2. Analysis of volatile organic compounds

The solid-phase microextraction head was inserted into the gas chromatography inlet under nitrogen protection and aged at 250°C for 0.5 h to remove the residual impurities. VOCs were extracted by headspace solid-phase microextraction. Casing layer samples (2 g) were added into a 20 mL headspace vial and extracted at 80°C for 45 min after equilibration for 10 min. After extraction, the volatiles were desorbed at 280°C for 5 min for identification and quantification in a gas chromatograph coupled with a mass spectrometer (Agilent 7890A/5975CGC-MS Agilent Technologies Inc., Santa Clara, CA, USA). The absorbed compounds were separated using a DB-5 ms capillary column (30 m × 0.25 mm, 0.25 μm). The GC oven program started at 40°C for 3 min, increased to 150°C at 5°C min^−1^ for 1 min and then to 240°C at 10°C min^−1^ for 5 min and ended at 260°C for 2 min. The flow rate of the carrier gas, helium, was 1.0 mL min^−1^. Split less injection was conducted. The ionization energy for MS was 70 eV in the electron impact mode. The temperature of the MS source was 230°C. The mass spectrum range was from 35 to 550 mass units. VOCs were identified by comparison with NIST/EPA/NIH (the 6th version) and the NIST 98 Mass Spectrometry Library from Wiley.

### 2.3. DNA extraction, amplification and sequencing

Genomic DNA was extracted from casing layer samples (2 g) using the FastDNA Kit for soil (MP bio, USA) according to the manufacturer’s instructions. The V3-V4 hyper-variable region of the bacterial 16S rRNA gene was amplified using the forward primer F (5′- CCTAYGGGRBGCASCAG-3′) and the reverse primer R (5′- GGACTACNNGGGTATCTAAT-3′) in a polymerase chain reaction (PCR) (ABI GeneAmp 9,700). PCR was performed in a 25 μL reaction mixture containing 1 × PCR buffer, 0.2 mM dNTPs, 0.2 M each primer, 0.6 Units TransStarFastpfu DNA polymerase (Axygen, USA) and 10 ng template DNA. The reaction cycling parameters were: 95°C for 2 min, 25 cycles of 95°C for 30 s, 55°C for 30 s and 72°C for 30 s, and a final extension at 72°C for 5 min. PCR was repeated three times for each DNA sample. The resulting products were pooled together, separated by electrophoresis on a 2% agarose gel, and purified with an AxyPrep DNA gel extraction kit (Axygen, USA). The purified DNA was quantified using the QuantiFluor™-ST fluorescence quantitative system (Promega, USA). The purified were subjected to sequencing on the Illumina MiSeq PE250 sequencing platform (Majorbio Bio-Pham Technology Co. Ltd., Shanghai, China).

### 2.4. Sequencing data processing

The reads in the dataset were clustered into operational taxonomic units (OTUs) using QIIME. Extracting non-repetitive sequences from optimized sequences can reduce the amount of redundant computation in the process of analysis then removing unduplicated sequences and the OTUs were assigned at a 97% identity threshold, and representative sequences for each OTU were classified against the Silva 16 s rRNA gene database (Quast et al., 2012). For fairly comparing the alpha and beta diversities in different samples, we selected the minimum number of sequences in all samples as the standard for normalization. ‘Aligner’ and ‘Complete Linkage Clustering’ were applied to calculate richness and diversity indices, namely the Chao Index, Simpson Index and Shannon Index. Principal component analysis (PCA), a multivariate statistical method that can be applied to reduce the dimensionality of variables and maximize the visible variability of the data, was used to estimate heterogeneity in microbial community composition among different casing layer samples.

### 2.5. Statistical analysis

F-test was performed to determine statistically significant differences between specific VOCs and different casing layers using SPSS (Version22). The Pearson indices, Spearman indices and *p* values for different parameters were calculated using R (v.3.0.2). The results of correlation analysis between microorganisms and yield are shown in the heat map, in which data are shown in a two-dimensional matrix and represented by different colors.

## 3. Results

### 3.1. Mushroom yield in different casing layer

**Figure 1:**
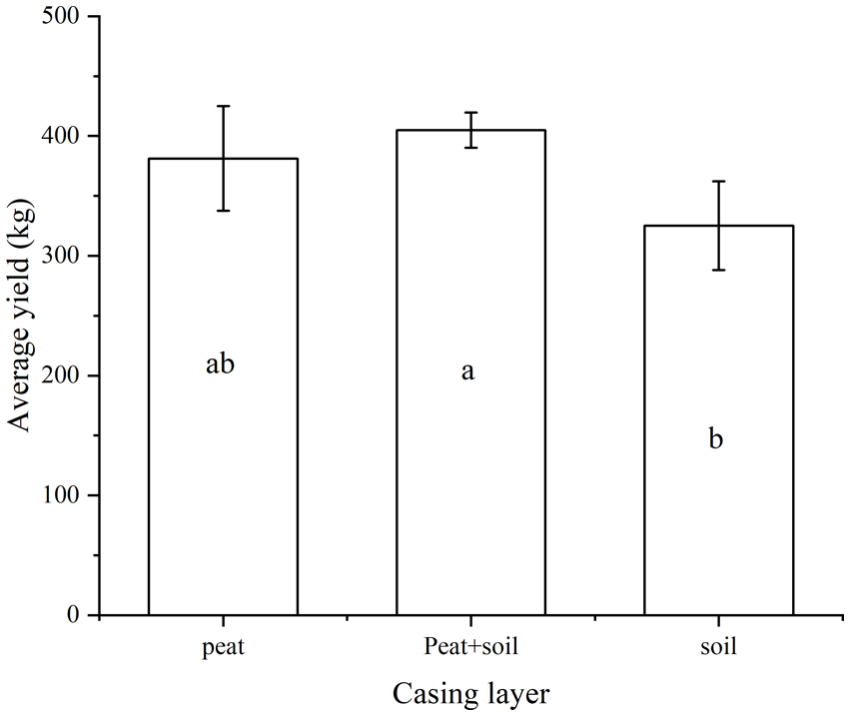
Comparison of the effects of different casing layer on the yield.

We examined the yield of *Agaricus bisporus* in different casing layers. The peat + soil and peat casing layers produced a higher yield of mushrooms as compared with the soil casing layer (*p* < 0.05, Figure 1). This result confirms that peat + soil is one of the best casing layer options. Peat also produced a similar yield as peat + soil, and peat + soil can obviously reduce the consumption of non-renewable peat.

### 3.2. Community richness varies but community diversity stays the same between casing layers

**Figure 2:**
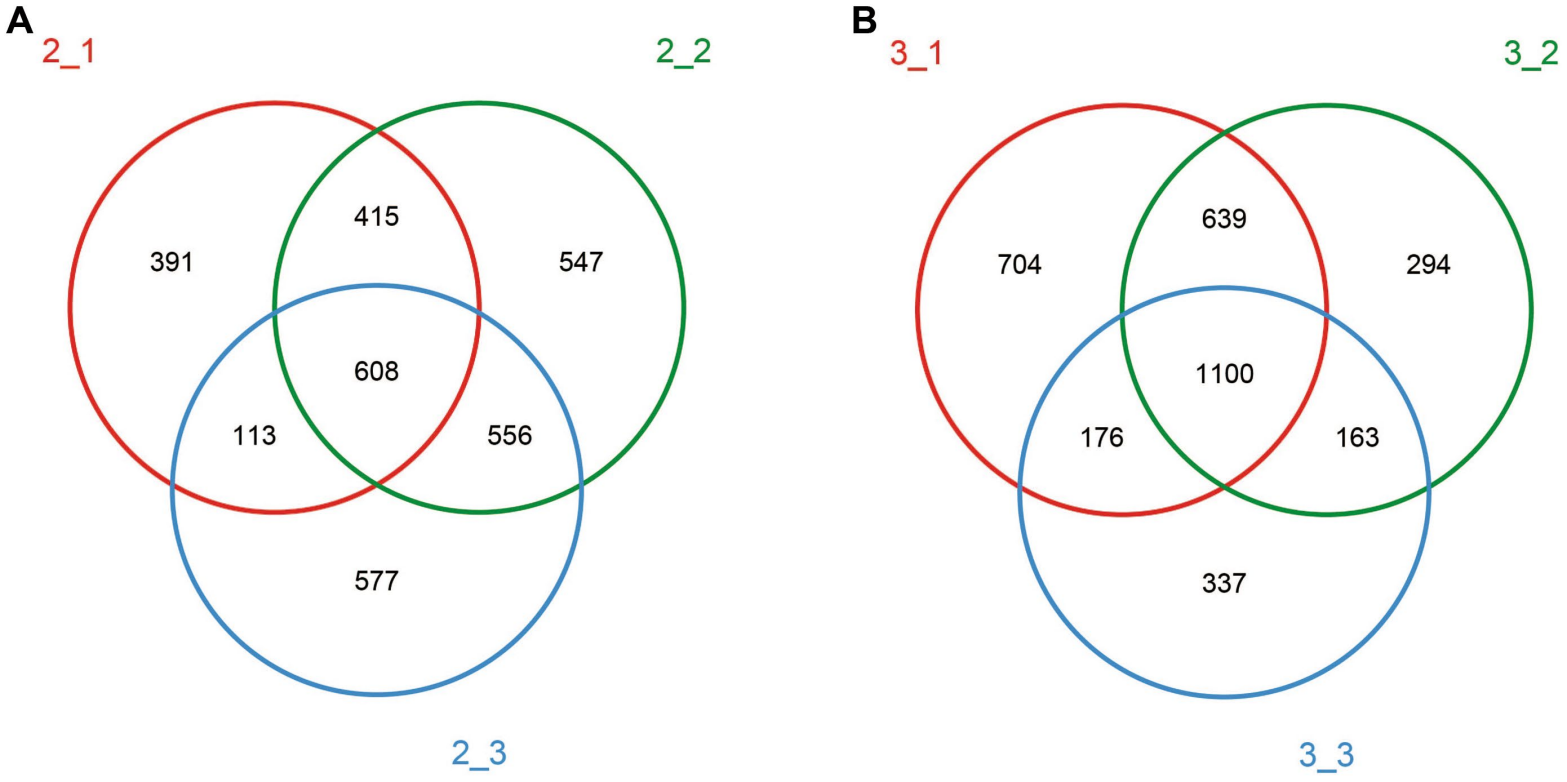
Venn diagram for different casing layers showing shared and unique OTU numbers. Different color represented different casing layers. Red: peat; green: peat + soil; blue: soil. **(A)** stage 1, **(B)** stage 3.

We detected 3,207 and 3,413 OTUs at stage 1 and stage 3, respectively, and 97% of the species were found in both stages (Figure 2). With respect to the similarities between casing layers, 608 OTUs (18.96%) at stage 1 and 1,100 OTUs (32.23%) at stage 3 were found in all three casing layers (Figure 2), which suggests that the similarity in microbial community composition between the three casing layers increases with increasing cropping stage. In addition, the similarity in OTUs between different samples, peat vs. peat + soil; peat + soil vs. soil; or soil vs. peat, also showed a trend of increase when comparing stage 1 to stage 3: 1023 (31.90%) vs. 1739 (50.95%), 1,164 (36.30%) vs. 1,263 (37.01%), or 721 (22.48%) vs. 1,276 (37.39%), respectively.

**Figure 3:**
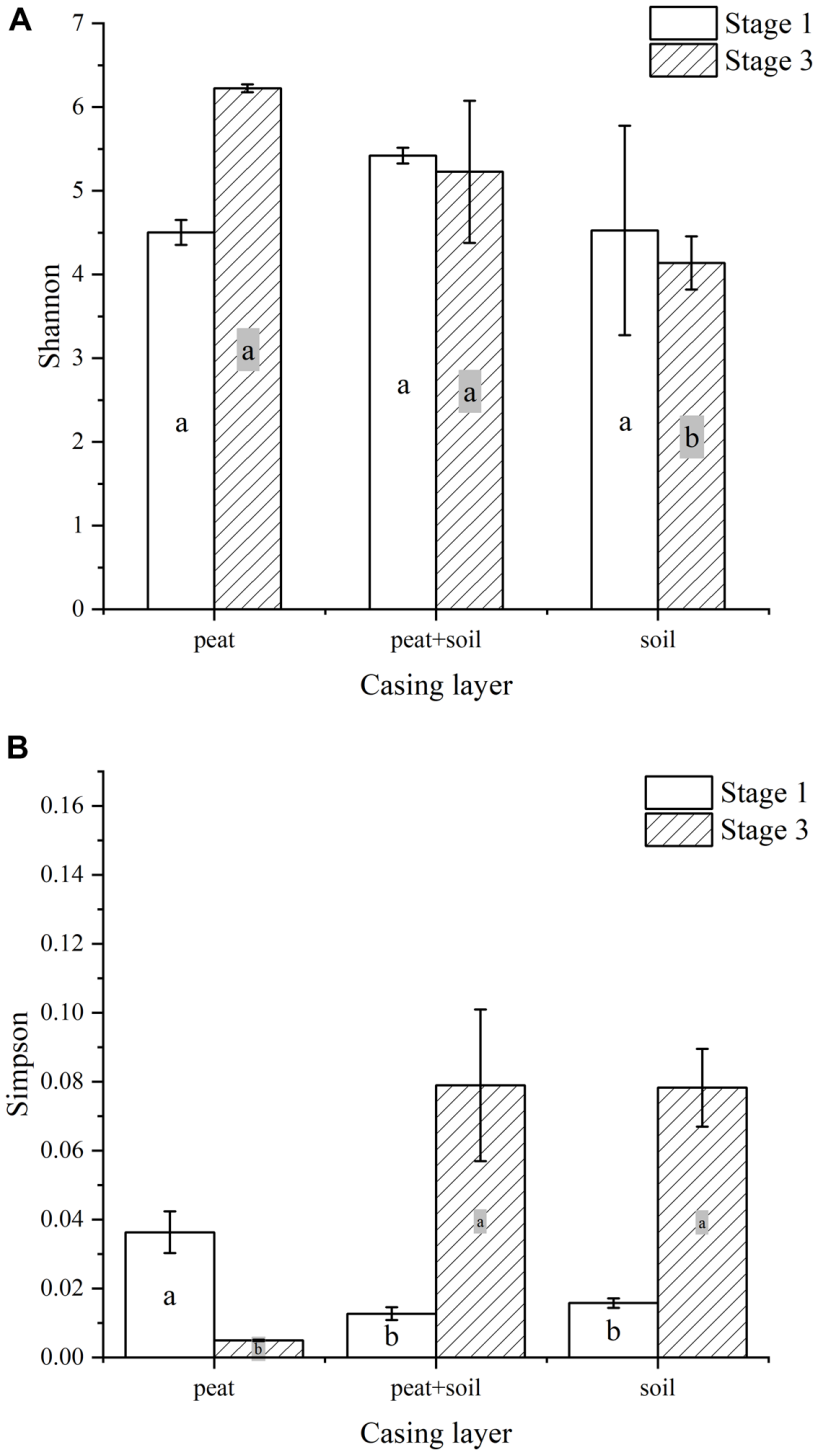
Shannon and Simpson curves at different casing layers. **(A)** Shannon, **(B)** Simpson.

Different casing layers also exhibited differences in community richness. At stage 1, the peat + soil samples exhibited the highest community richness (OTUs 1,660, chao 2,117), followed by the soil samples (OTUs 1,228, chao 1,653) and then peat samples (OTUs 1,149, chao 1,582). In contrast, at stage 3, the peat samples exhibited the highest community richness (OTUs 2,371, chao 3,151), followed by peat + soil (OTUs 1,393, chao 1750) and soil (OTUs 1,124, chao 1,438) (Table 1). The community diversity did not differ between three casing layers at the stage 1 based on the Shannon index (*p* > 0.05, Figure 3A). But the peat and peat + soil casing layers produced a higher diversity as compared with the soil casing layer (*p* < 0.05, Figure 3A) The differences in community diversity between casing layers are statistically significant based on the Simpson index, with the highest diversity observed in peat samples at stage 1 and the higher diversity observed in the peat and peat + soil samples (*p* < 0.05, Figure 3B).

**Table 1:** Comparison of community richness and community diversity estimators of bacterial communities in different casing layer samples at stage 1 and stage 3.

Stage	Sample ID	Reads	0.97					
			OTU	Ace	Chao	Coverage	Shannon	Simpson
Stage 1	2_1_1	49,820	1,238	1,657 (1,579,1753)	1,649 (1,553,1773)	0.992272	4.66 (4.64,4.68)	0.0295 (0.0289,0.0301)
	2_1_2	32,883	1,019	1724 (1,627,1837)	1,440 (1,340,1,570)	0.988688	4.36 (4.34,4.38)	0.041 (0.04,0.042)
	2_1_3	43,595	1,189	1,625 (1,544,1726)	1,658 (1,549,1800)	0.990962	4.49 (4.47,4.51)	0.0385 (0.0376,0.0393)
	2_2_1	57,059	1838	2,314 (2,235,2,408)	2,295 (2,201,2,413)	0.991237	5.36 (5.35,5.38)	0.0147 (0.0145,0.015)
	2_2_2	42,246	1,407	1861 (1779,1961)	1922 (1806,2071)	0.989940	5.37 (5.36,5.38)	0.011 (0.0108,0.0112)
	2_2_3	49,450	1735	2,164 (2091,2,253)	2,133 (2048,2,240)	0.990758	5.53 (5.51,5.54)	0.0125 (0.0122,0.0128)
	2_3_1	32,670	784	1,482 (1,381,1,600)	1,251 (1,129,1,415)	0.990266	3.09 (3.07,3.12)	0.01617 (0.01584,0.01649)
	2_3_2	49,677	1,407	1806 (1732,1896)	1814 (1721,1936)	0.992069	5.12 (5.1,5.14)	0.017 (0.0167,0.0173)
	2_3_3	37,188	1,492	1933 (1856,2027)	1894 (1806,2006)	0.988249	5.37 (5.35,5.38)	0.0143 (0.0139,0.0147)
Stage 3	3_1_1	55,647	2,538	3,367 (3,253,3,499)	3,412 (3,262,3,595)	0.986253	6.24 (6.22,6.25)	0.005 (0.0049,0.0051)
	3_1_2	41,974	2,452	3,909 (3,769,4,064)	3,372 (3,219,3,556)	0.980464	6.26 (6.25,6.28)	0.0048 (0.0047,0.0049)
	3_1_3	54,599	2,123	2,591 (2,514,2,682)	2,669 (2,558,2,809)	0.990476	6.17 (6.16,6.19)	0.0052 (0.0051,0.0053)
	3_2_1	29,852	1,351	1,669 (1,607,1745)	1,692 (1,610,1745)	0.988275	5.66 (5.65,5.68)	0.084 (0.082,0.087)
	3_2_2	45,685	1864	2,308 (2,234,2,396)	2,328 (2,232,2,450)	0.989274	5.77 (5.76,5.79)	0.098 (0.095,0.1)
	3_2_3	40,765	964	1,282 (1,215,1,367)	1,231 (1,162,1,325)	0.992812	4.25 (4.23,4.27)	0.0549 (0.0535,0.0562)
	3_3_1	53,770	1,361	1,699 (1,634,1779)	1732 (1,644,1847)	0.993193	4.39 (4.37,4.41)	0.0659 (0.0644,0.0673)
	3_3_2	43,888	777	1,012 (956,1,083)	984 (925,1,068)	0.994759	3.78 (3.76,3.8)	0.0881 (0.0862,0.0901)
	3_3_3	38,172	1,235	1,576 (1,510,1,657)	1,598 (1,512,1712)	0.990569	4.24 (4.22,4.27)	0.0808 (0.0787,0.0828)

### 3.3. The microbial community is very dynamic

**Figure 4:**
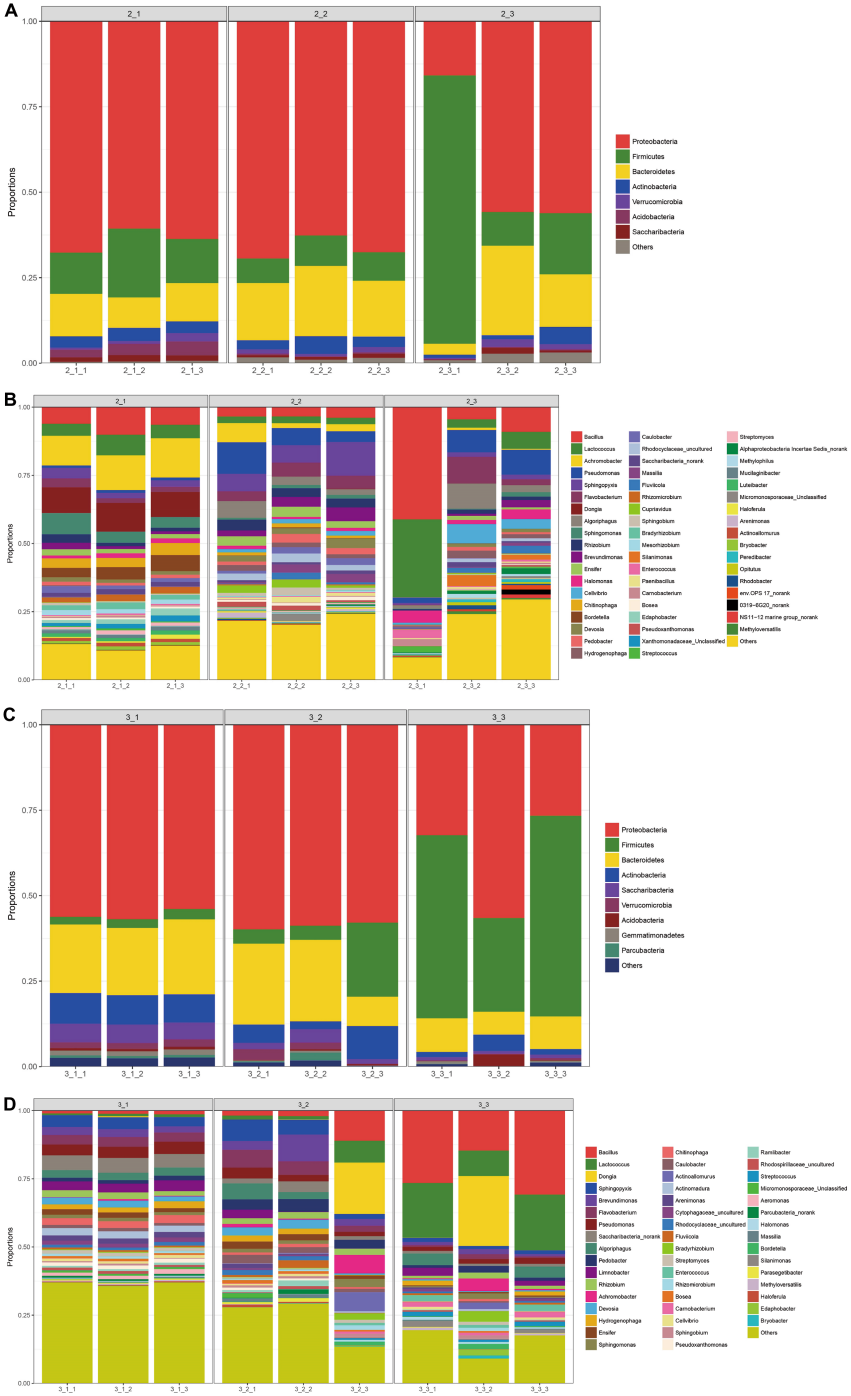
The bacterial community composition at the phylum level and genus level. The category “others” denoted that with relative abundance <1%. **(A)** at the phylum level of stage 1, **(B)** at the genus level of stage 1, **(C)** at the phylum level of stage 3, **(D)** at the genus level of stage 3.

We next examined the microbial community at both phylum and genus levels. As shown in Figures 4A,C, at phylum level, *Proteobacteria* predominated in all casing layers at the two stages, which was similar to the finding that *Proteobacteria* is the most abundant phylum in other microbial communities from different environments such as soil (Roesch et al., 2007) and sewage (Zhang et al., 2012). The second and third most abundant phyla were *Firmicutes* and *Bacteroidetes*, respectively, and the relative abundance of the top three phyla combined was over 75%. In addition, soil had the highest abundance of *Firmicutes* and lowest abundance of *Proteobacteria* when compared with the other two casing layers, especially at stage 3.

At the genus level, the microbial communities in the different casing layers became more divergent, even though a similar number of genera, 674 and 675, were identified at stage 1 and stage 3, respectively (Figures 4B,D). The dominant bacteria at stage I were *Bacillus*, *Lactococcus*, *Achromobacter*, *Pseudomonas*, *Sphingopyxis*, *Flavobacterium* and *Dongia*. In contrast, the primary genera of bacteria at stage 3 were *Bacillus*, *Lactococcus*, *Sphingopyxis*, *Brevundimonas*, *Flavobacterium* and *Pseudomonas*.

**Figure 5:**
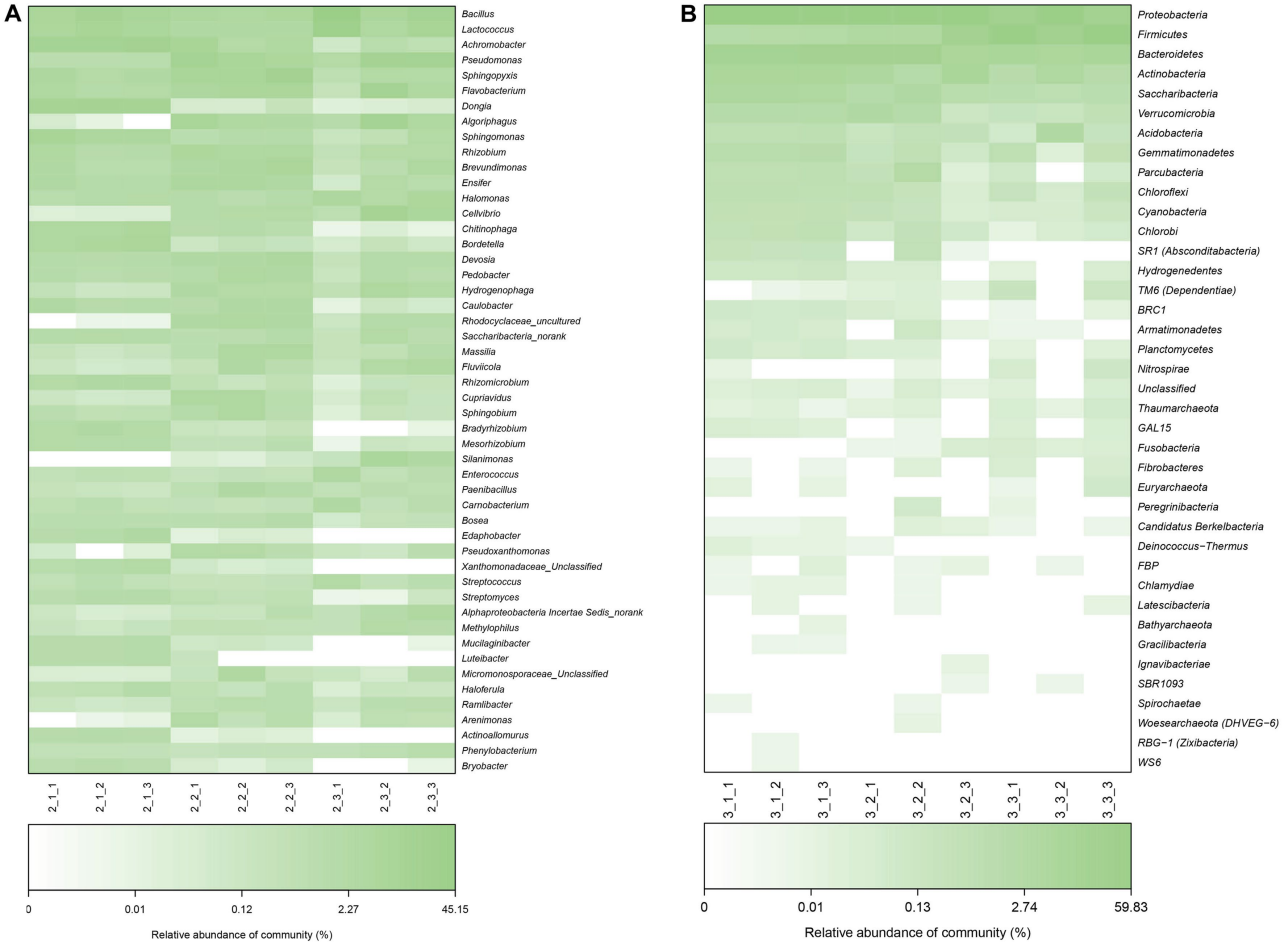
Heatmap analysis of microbial communities based on the Bray–Curtis similarity at genus level (top 50) at stage 1 and stage 3. **(A)** stage 1, **(B)** stage 3.

We plotted a hierarchical heat map for the top 50 abundant bacterial communities at the genus level and classified these genera into four groups according to their abundance in different casing layers at stage 1 (Figure 5). The bacteria in the first group were most abundant in peat samples and include *Achromobacter*, *Dongia* and *Sphingomonas*. The second groups of bacteria were most abundant in peat + soil or soil samples and include *Pseudomonas*, *Algoriphagus* and *Cellvibrio*. Bacteria in the third group had nearly the same abundance in all three casing layers at stage 3.

### 3.4. The microbial community varies between casing layers and cropping stages

**Figure 6:**
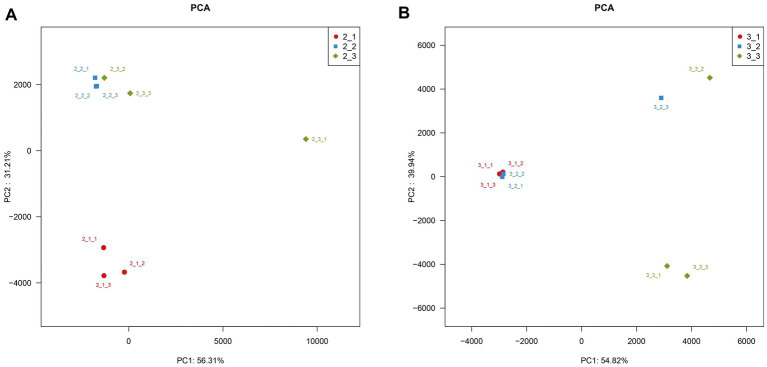
PCA analysis to estimate heterogeneity in microbial community composition among different samples. **(A)** stage 1, **(B)** stage 3.

Our results indicate that microbial community diversity significantly between different casing layers at different cropping stages (*p* < 0.05, Figure 3). Moreover, the three casing layers had higher similarities in microbial community composition at the later cropping stage (Figure 2). We further examined the sequencing data using PCA, a method used to estimate heterogeneity in microbial community composition among different samples. We did not observe a significant overlap in PCA values among the three casing layers (Figure 6), which indicates that the microenvironment created by a specific casing layer can significantly remodel the microbial community structure. We also noticed that the distances between samples from peat + soil and soil were closer than those from peat in the PCA scatter plot at stage 1 (Figure 6). In contrast, samples from peat and peat + soil were clustered more closely together than those from soil at stage 3 (Figure 6). This suggests that microbial community structure also differs between cropping stages.

### 3.5. Specific microorganisms are involved in yield formation

**Figure 7:**
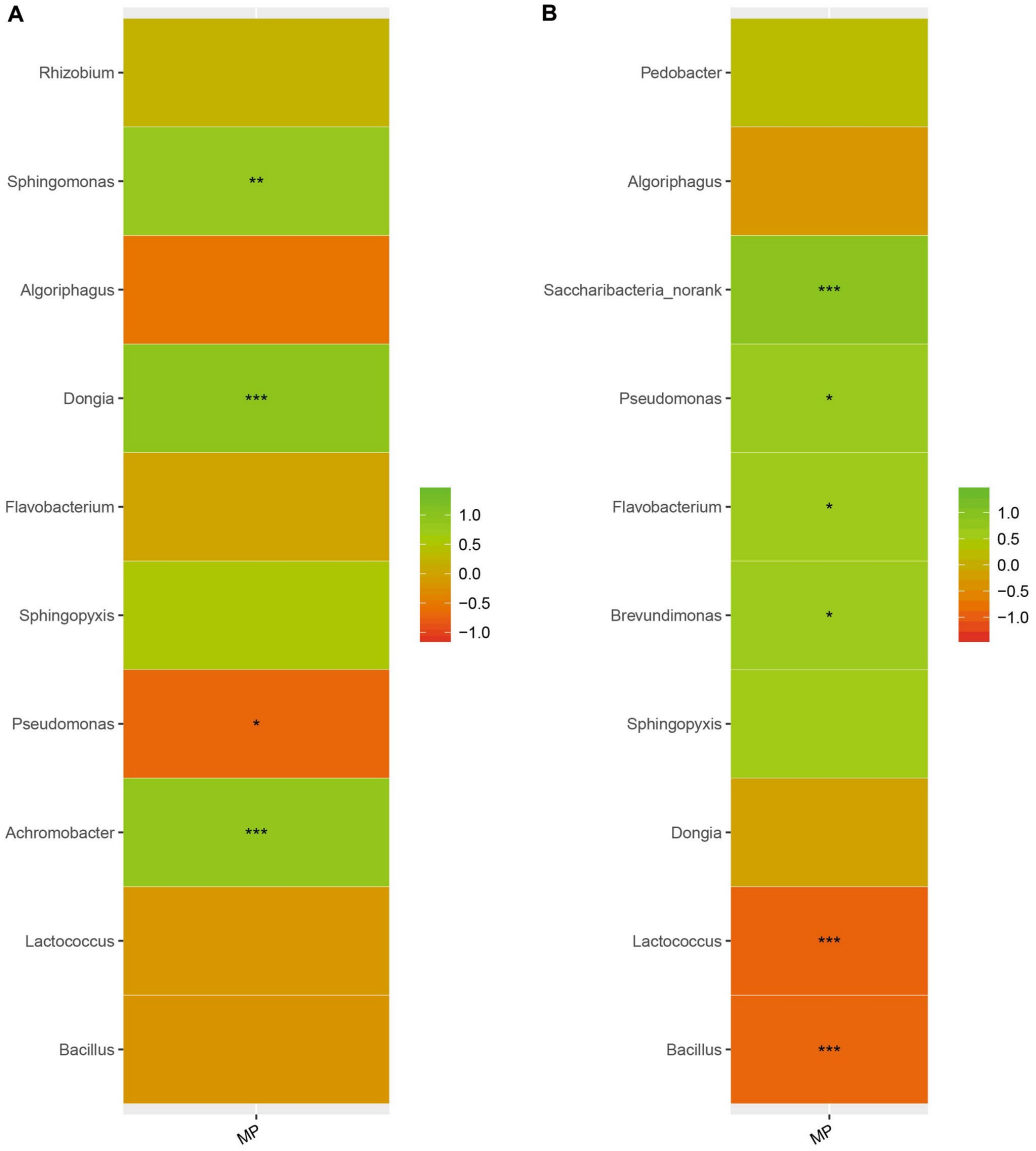
The relationship between microorganisms and yield. Different colors indicate different groups. On the middle is the *p* value. *0.01 < *p* ≤ 0.05, **0.001 < *p* ≤ 0.01, *** *p* ≤ 0.001. **(A)** stage 1, **(B)** stage 3.

We analyzed the correlation between microorganisms in the casing layers at stage 1 and stage 3 and the final mushroom yield. Microorganisms were strongly correlated with yield. We found that *Sphingomonas*, *Dongia* and *Achromobacter* were positively correlated with yield at stage 1, while *Saccharibacteria_norank*, *Pseudomonas*, *Flavobacterium* and *Brevundimonas* were positively correlated with yield at stage 3. In contrast, *Pseudomonas* was negatively correlated with yield at stage 1, and *Lactococcus* and *Bacillus* were negatively correlated with yield at stage 3 (Figure 7). These results suggest that mushroom yield can be affected by adding specific microorganisms.

### 3.6. Specific VOCs in the casing layer at different cropping stages

**Figure 8:**
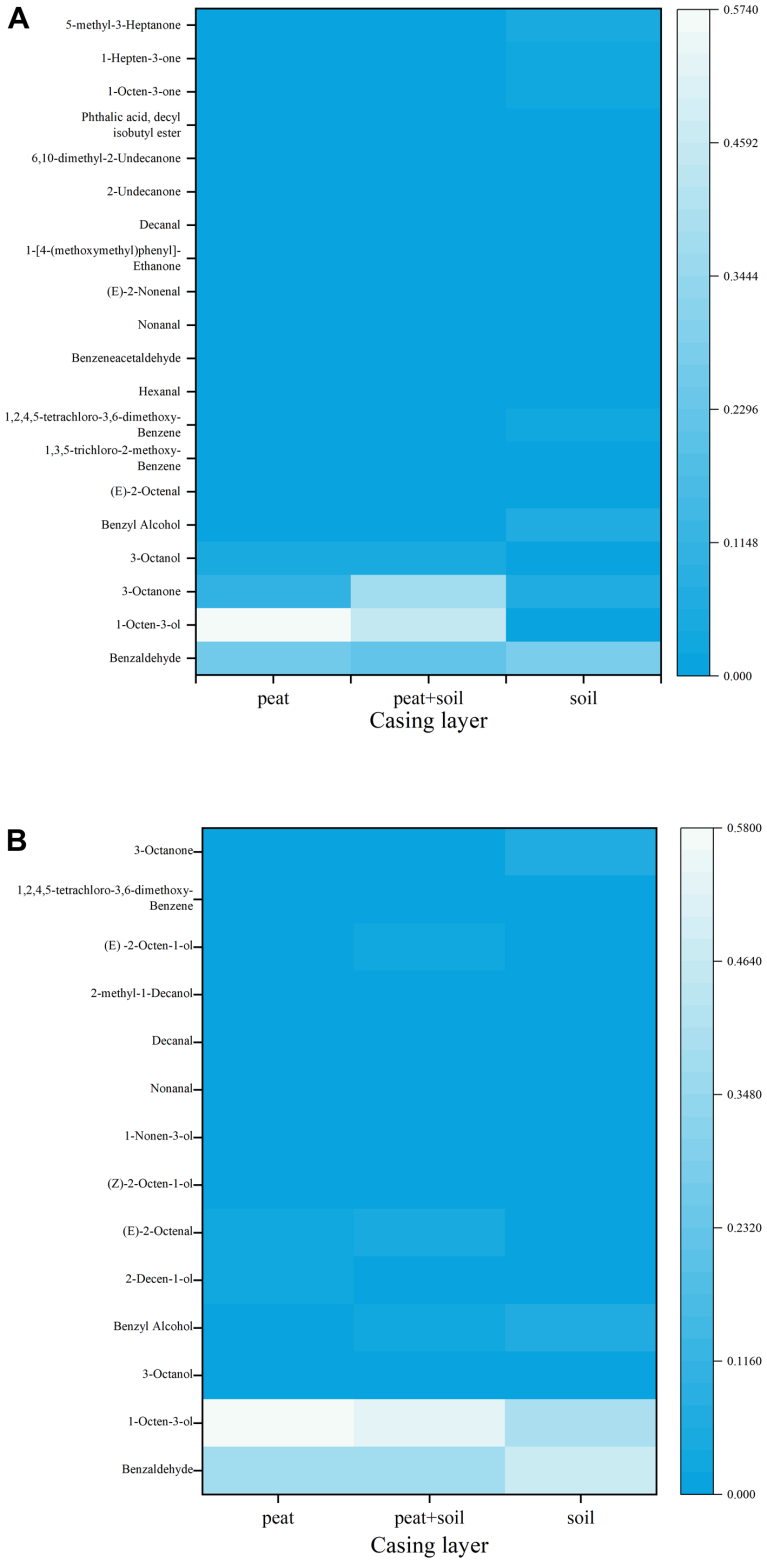
Heatmap analysis of VOCs at different casing layers. **(A)** stage 1, **(B)** stage 3.

**Figure 9:**
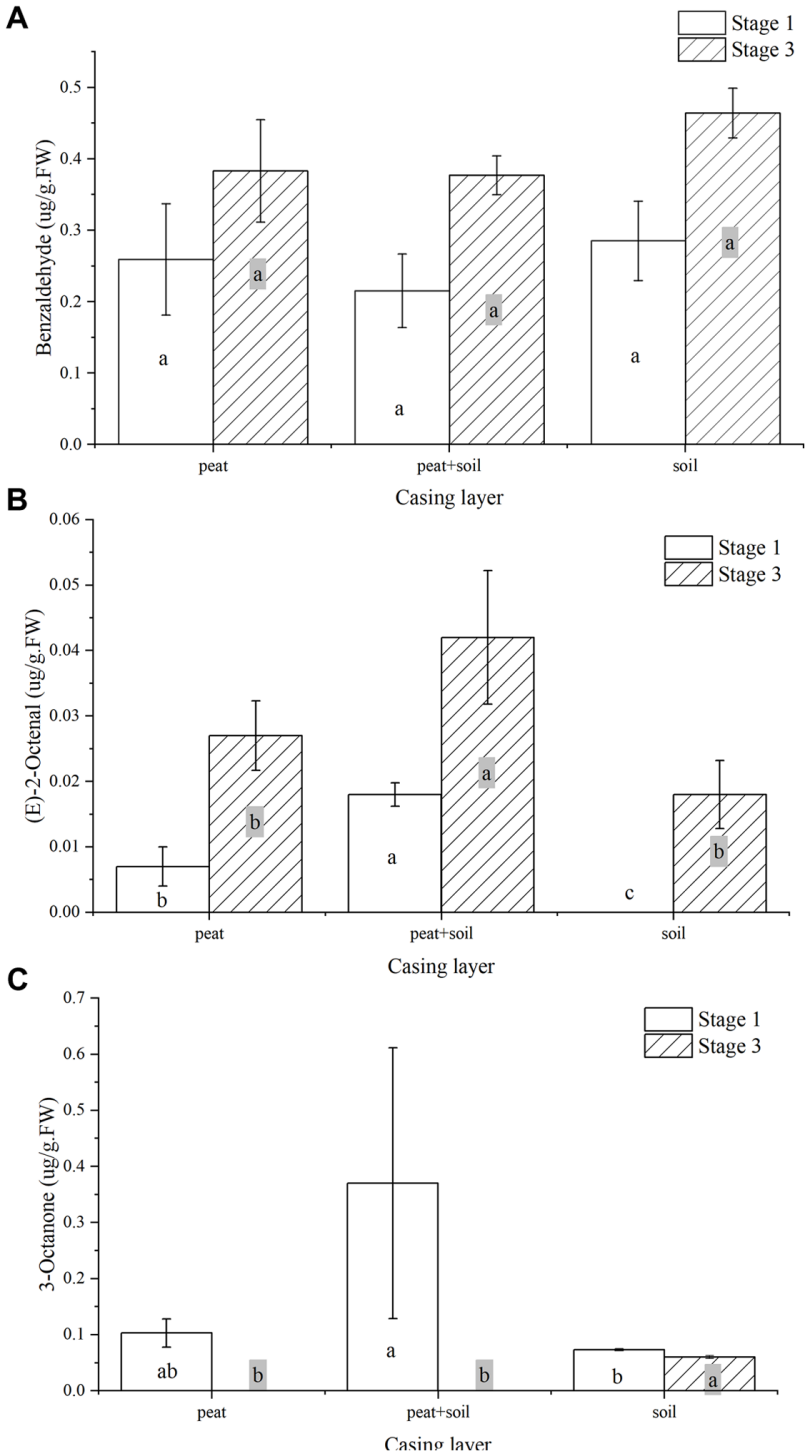
The change of benzaldehyde, **(E)**-2-octenal, and 3-octanone in all three casing layers during the developmental transition from stage 1 to stage 3. **(A)** benzaldehyde, **(B)** (E)-2-octenal, **(C)** 3-octanone.

We identified 8, 15 and 7 VOCs in peat, peat + soil and soil, respectively, at stage 1. The compounds found in all three casing layers were benzaldehyde, 3-octanone and 1,2,4,5-tetrachloro-3,6-dimethoxy-benzene (Table 2; Figure 8). Among these VOCs, benzaldehyde, 1-octen-3-ol and 3-octanone compounds were abundant in peat and peat + soil while benzaldehyde was plentiful in soil. We also identified 11, 8 and 7 VOCs in peat, peat + soil and soil, respectively, at stage 3, among which benzaldehyde, 1-octen-3-ol, benzyl alcohol and (E)-2-octenal were abundant in all three casing layers, with the first two being the most abundant (Table 3; Figure 8). By comparing the differences between VOCs in stages 1 and 3, Benzaldehyde and (E)-2-octenal tended to increase in all three casing layers and 3-octanone tended to decrease during the developmental transition from stage 1 to stage 3 (Figure 9). These observations indicate that benzaldehyde and (E)-2-octenal promote the formation of the sporophore while 3-octanone inhibits it. However, we did not see a correlation between VOCs and final yield.

**Table 2:** Volatile organic compounds on mushroom production at stage 1.

Casing layer	VOCs	RT (min)	Concentration (μg g^−1^ FW)
Peat	Benzaldehyde	10.541	0.259 ± 0.078
	1-Octen-3-ol	11.361	0.574 ± 0.038
	3-Octanone	11.456	0.103 ± 0.025
	3-Octanol	11.769	0.040 ± 0.014
	Benzyl Alcohol	12.878	0.012 ± 0.002
	(E)-2-Octenal	13.661	0.007 ± 0.003
	1,3,5-trichloro-2-methoxy- Benzene	21.497	0.002 ± 0.0002
	1,2,4,5-tetrachloro-3,6-dimethoxy- Benzene	30.252	0.004 ± 0.0005
Peat + soil	Hexanal	5.607	0.011 ± 0.0025
	Benzaldehyde	10.569	0.215 ± 0.0516
	1-Octen-3-ol	11.394	0.444 ± 0.0413
	3-Octanone	11.489	0.370 ± 0.2415
	3-Octanol	11.773	0.040 ± 0.0170
	Benzeneacetaldehyde	13.183	0.009 ± 0.0016
	(E)-2-Octenal	13.677	0.018 ± 0.0018
	Nonanal	15.137	0.007 ± 0.000009
	(E)-2-Nonenal	16.802	0.002 ± 0.0001
	1-[4-(methoxymethyl)phenyl]- Ethanone	17.676	0.003 ± 0.0002
	Decanal	18.183	0.004 ± 0.003
	2-Undecanone	20.627	0.004 ± 0.007
	6,10-dimethyl-2-Undecanone	23.574	0.005 ± 0.0009
	1,2,4,5-tetrachloro-3,6-dimethoxy- Benzene	30.252	0.005 ± 0.0006
	Phthalic acid, decyl isobutyl ester	32.259	0.001 ± 0.000004
Soil	Benzaldehyde	10.512	0.285 ± 0.0557
	1-Octen-3-one	11.044	0.034 ± 0.0094
	1-Hepten-3-one	11.139	0.036 ± 0.0038
	5-methyl-3-Heptanone	11.328	0.049 ± 0.0033
	3-Octanone	11.349	0.073 ± 0.0013
	Benzyl Alcohol	12.862	0.060 ± 0.0195
	1,2,4,5-tetrachloro-3,6-dimethoxy- Benzene	30.252	0.020 ± 0.0057

**Table 3:** Volatile organic compounds on mushroom production at stage 3.

Casing layer	VOCs	RT (min)	Concentration (μg g^−1^ FW)
Peat	Benzaldehyde	10.706	0.383 ± 0.0717
	1-Octen-3-ol	11.468	0.580 ± 0.0882
	3-Octanol	11.365	0.005 ± 0.0002
	Benzyl Alcohol	12.445	0.010 ± 0.0072
	2-Decen-1-ol	13.558	0.026 ± 0.0035
	(E)-2-Octenal	13.715	0.027 ± 0.0053
	(Z)-2-Octen-1-ol	13.991	0.019 ± 0.0011
	1-Nonen-3-ol	14.395	0.003 ± 0.0002
	Nonanal	15.137	0.003 ± 0.0003
	Decanal	18.179	0.003 ± 0.0005
	2-methyl-1-Decanol	30.602	0.005 ± 0.0003
Peat + soil	Benzaldehyde	10.607	0.377 ± 0.0272
	1-Octen-3-ol	11.419	0.538 ± 0.0283
	Benzyl Alcohol	12.907	0.029 ± 0.0008
	(E)-2-Octenal	13.69	0.042 ± 0.0102
	(E) -2-Octen-1-ol	13.987	0.027 ± 0.0087
	1-Nonen-3-ol	14.395	0.003 ± 0.0006
	Nonanal	15.137	0.003 ± 0.0002
	1,2,4,5-tetrachloro-3,6-dimethoxy- Benzene	30.248	0.003 ± 0.0008
Soil	Benzaldehyde	10.586	0.464 ± 0.0347
	1-Octen-3-ol	11.262	0.400 ± 0.0810
	3-Octanone	11.373	0.060 ± 0.0027
	3-Octanol	11.728	0.012 ± 0.0053
	Benzyl Alcohol	12.878	0.058 ± 0.0337
	(E)-2-Octenal	13.665	0.018 ± 0.0052
	(E)-2-Octen-1-ol	13.945	0.013 ± 0.0040

### 3.7. Verification testing

**Figure 10:**
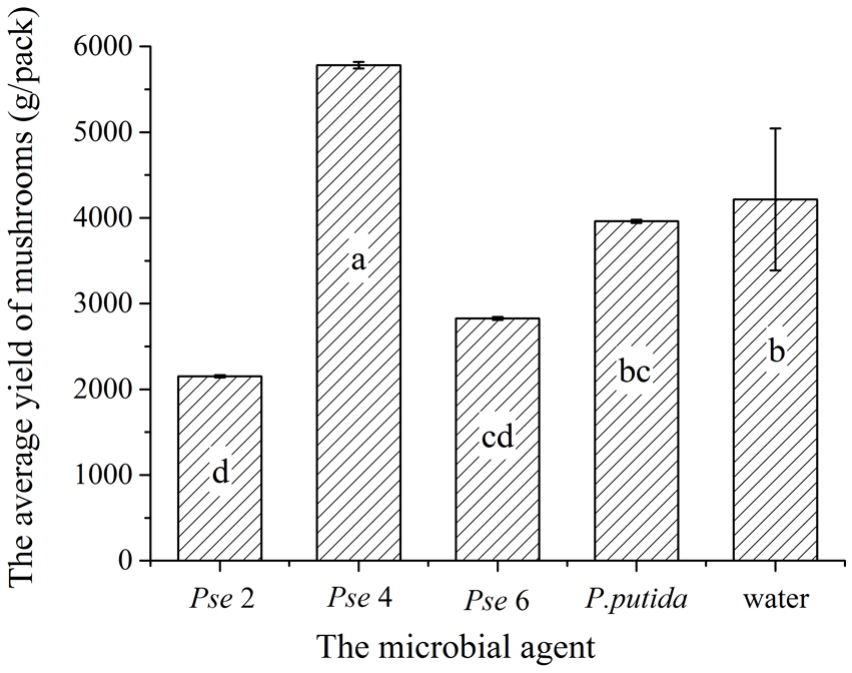
Verification testing.

Finally, we performed a verification experiment to manually spray different types and microbial agents during the kinking of *Agaricus bisporus*. The earliest buds were the control group, and the last buds were *Pseudomonas putida*. The mushroom buds were dense in the *Pseudomonas putida* group. The highest mushroom yield was produced by spraying four bottles of *Pseudomonas*; the total weight grown was 17.343 kg. The control group produced the second highest total yield at 14.649 kg (Figure 10). The lowest yield came from the test group, which received two bottles of *Pseudomonas*. The yield was only 6.455 kg. This experiment shows that the production of *A. bisporus* can be improved by adding microbial agents properly.

## 4. Discussion

*Agaricus bisporus* can occasionally develop on “uncased” beds, but casing is necessary to obtain a plentiful growth of sporophores (Lambert, 1938; Noble et al., 2003; Zhang et al., 2023). However, the precise manner in which the casing layer stimulates mushroom development is still not well determined. Previous studies mainly focused on the physical and chemical properties of casing layers (Noble et al., 1999; Beyer et al., 2002; Pardo et al., 2010), but volatile compounds and microorganisms have been poorly studied (Hayes et al., 1969; Noble et al., 2009). The VOCs secreted by the *A. bisporus* mycelium are mainly 1-Octen-3-ol and ethylene (Meng et al., 2014; Zhang et al., 2016; Carrasco and Preston, 2020). Zhang et al. (2023) make a review on the synthesis and action mechanism of 1-Octen-3-ol and ethylene for mushroom formation. Besides, Zhang et al. (2022) reviews the synthesis pathway, function and the mechanism of inhibiting the mushroom formation of the two volatile compounds and think the volatile self-inhibitor synthesized by the mushroom mycelia is ethylene, not 1-octen-3-ol. Moreover, previous studies were conducted using a special apparatus, such as Petri dish, but not in actual practice, and the relationship of the compounds and microorganisms to yield was not determined. In this study, we conducted GC–MS and meta genomic analyses to systematically identify volatile compounds and microorganisms in three different casing layers at two mushroom cropping stages. We identified new volatile compounds in the casing layers (Tables 2, 3), although their effect on mushroom production needs to be further studied. The contents of benzaldehyde and (E)-2-octenal increased significantly and the content of 3-octanone decreased significantly during development from stage 1 to stage 3 (Figure 9). We also systematically characterized the microbial community structures in the three casing layers (Figure 4) and found a significant difference between casing layers (Figure 6). The most striking discovery is the association of specific microorganisms with yield in the actual production of *A. bisporus* (Figure 7). Those bacteria potentially promoting yield include *Sphingomonas*, *Dongia* and *Achromobacter* at stage 1 and *Saccharibacteria_norank*, *Pseudomonas*, *Flavobacterium* and *Brevundimonas* at stage 3, while those associated with reduced yield was *Pseudomonas* at stage 1 and by *Lactococcus* and *Bacillus* at stage 3.

Researchers conducted studies on volatile compounds obtained from sporophores, mycelium or substrate (Grove, 1981; Noble et al., 2009; Costa et al., 2013, 2015). We noticed that compounds such as 1-octen-3-ol, 3-octanol, 3-octanol, 2-octen-1-ol, 1-octen-3-one, (E)-2-octenal, benzyl alcohol, benzaldehyde and hexanal that were previously detected in *Agaricus bisporus*, were also found in different casing layers (Tables 2, 3). Of these compounds, the 8-carbon volatiles are characteristic constituents in fungi that provide unique mushroom odorants (Csóka et al., 2017). Benzaldehyde and benzyl alcohol were found to be the most abundant compounds in *A. bisporus* (Csóka et al., 2017). These common compounds must be produced by *A. bisporus* and then absorbed by the casing layer. We also identified some new volatile compounds such as 1,2,4,5-tetrachloro-3,6-dimethoxy-benzene.

1-octen-3-ol is the volatile compound with the greatest inhibitory activity against primordium formation (Wood and Blight, 1983; Noble et al., 2009). Although there was no significant alteration in 1-octen-3-ol content between stage 1 and stage 3 (Tables 2, 3), the amount of 1-octen-3-ol was significantly decreased compared with those of mycelia growth stage in three casing layers, which is consistent with the inhibitory effect of 1-octen-3-ol on primordium formation. The primordium had formed at stage 1 and stage 3, and 1-octen-3-ol stayed at the same level. The presence of volatile compounds, such as 2-ethyl-1-hexanol and 1-octen-3-ol, can also result in higher pseudomonad populations in the casing. The removal of inhibitory volatile compounds by microbes in the casing layer can stimulate primordium formation. Besides, other volatile compounds might play an important role in stimulating sporophore formation, such as benzaldehyde and (E)-2-octenal (Figure 9), although further studies are needed to test this. It is reasonable to believe that the increase in specific microbial richness would help to metabolize some inhibitory volatile compounds.

*Agaricus bisporus* are cultivated in fermented substrates where the microbiome plays a significant role in fungal growth and fructification (Vieira and Pecchia, 2018). A wide variety of interactions is established among bacteria and fungi ranging from antagonism and competition to mutualism during cultivation of mushrooms (Deveau et al., 2018). Two research teams found that bacteria in soil could stimulate sporophore formation (Hayes et al., 1969; Park and Agnihotri, 1969). Hayes et al. (1969) characterized the ability of individual bacteria isolates utilizing volatile metabolites to induce the fruiting of *A. bisporus*. They identified *Pseudomonas putida* as the primary bacterium stimulating sporophore initiation. Another study revealed that a diverse range of bacteria, including *Arthrobacter terregens*, *Bacillus megaterium* and *Rhizobium meliloti* and their metabolites could trigger sporophore formation in a Petri dish (Park and Agnihotri, 1969). Related studies focusing on *Pseudomonas* (Rainey et al., 1990; Noble et al., 2009) discovered that the ability of different *Pseudomonas* isolates to stimulate primordium initiation varied. The abundance of Proteobacteria, particularly *Pseudomonas*, with a corresponding decrease in *Firmicutes* and *Actinobacteria* in the casing layer was found to increase during the growth of *A. bisporus* (Hayes and Nair, 1974; Carrasco and Preston, 2020). One potential explanation for this is that the consumption of 1-octen-3-ol by *Pseudomonas* spp. may promote mushroom fructification (Carrasco et al., 2019). Researchers also found that although axenic casing inoculated with different *Pseudomonas* isolates could induce primordial initiation, the number of primordia was obviously lower than that non-axenic casing (Noble et al., 2009), which suggests that a mixed *Pseudomonas* or bacterial population is more efficient than a single isolate in stimulating primordium formation (Eger, 1972; Noble et al., 2009). Here, we analyzed the microbial community in three different casing layers and discovered more potentially valuable microorganisms for mushroom production.

The results demonstrate that the bacterial community in the casing layer is mainly comprised of *Proteobacteria*, *Firmicutes* and *Bacteroidetes* at the phylum level (Figures 4A,C), which is consistent with the composition found in previous studies in soil (Li et al., 2018; Wu et al., 2018; Carrasco and Preston, 2020). At the genus level, we recorded different dominant bacterial genera (Figures 4B,D) in different casing layers at different cropping stages, which seem to indicate that the type of casing layer and the mushroom growth process can affect the bacterial community. Our discoveries are consistent with the reported dominance of *Pseudomonas*, *Flavobacterium*, *Proteobacterium* and uncultured species in casing materials analyzed using denaturing gradient gel electrophoresis (Siyoum et al., 2010).

The correlation analysis indicates that some specific microorganisms have a positive or negative relationship with the final yield (Figure 7). Han (1999) reported that spraying casing with a suspension of photosynthetic bacteria could significantly increase the yield of *Agaricus bisporus*. Some bacteria associated with the casing layer could initiate significantly more basidiocarps than other bacteria when added to the casing (Reddy and Patrick, 1990). Two indigenous mushroom growth-promoting bacteria *Pseuodomonasputia* strains Bt4 and Ps7, increased mushroom yield by about 14% when inoculated in the casing layer (Zarenejad et al., 2012).

The presence of volatile compounds, such as 2-ethyl-1-hexanol and 1-octen-3-ol, can also result in higher pseudomonad populations in the casing. The removal of inhibitory volatile compounds by microbes in the casing layer can stimulate primordium formation (Noble et al., 2009). Therefore, it is reasonable to believe that the increase in specific microbial richness would help to metabolize some inhibitory volatile compounds.

## 5. Conclusion

In conclusion, by utilizing three different casing layers corresponding to different mushroom yields, our study systematically evaluated the contents of volatile compounds and microbial community structure in different casing layers at different cropping stages. To the best of our knowledge, this is the first study in which high throughput sequencing was used to analyze the microbial community in casing layers. More importantly, we evaluated the relationship of specific volatile compounds and microorganisms with the yield of *Agaricus bisporus*. Benzaldehyde and (E)-2-octenal promote the formation of the sporophore while 3-octanone inhibits it. However, we did not see a strong relationship between volatile compounds and yield, but we found that specific microorganisms, including *Sphingomonas*, *Dongia* and *A chromobacter* at stage 1, *Saccharibacteria_norank, Pseudomonas*, *Flavobacterium* and *Brevundimonas* at stage 3 were positively correlated with yield, while *Pseudomonas* at stage 1 and *Lactococcus* and *Bacillus* at stage 3 were negatively correlated with yield. Overall, we propose that it is necessary to determine the best method for application of specific bacteria identified in our study that are positively correlated with yield at stage 1 or stage 3. As far as volatile compounds are concerned, it is not recommended to increase production by increasing them. Our results provide a guide for the development and agricultural application of microbial agents for yield improvement in the production of *A. bisporus*.

## Data availability statement

The data presented in the study are deposited in the NCBI, accession number PRJNA967167.

## Author contributions

P-FR and X-YY helped design this study and performed the statistical analysis. P-FR carried out the study, collected important background information, and drafted the manuscript. P-FR, Y-HW, X-YY, and L-ZW are responsible for the concepts, design, definition of intellectual content, literature search, data acquisition, data analysis, and manuscript preparation. H-XR and LQ helped with data acquisition, data analysis, and statistical analysis. H-DG, L-LD, and XL performed the literature search, acquired data, and edited the manuscript. Y-HW, X-YY and XL reviewed the manuscript. All authors have read and approved the content of the manuscript.

## Funding

This work is supported by China Agriculture Research System-20 (CARS-20), Major innovation project of Shandong Province (2022CXGC010610), and the National Natural Science Foundation of China (32200014).

## Conflict of interest

The authors declare that the research was conducted in the absence of any commercial or financial relationships that could be construed as a potential conflict of interest.

## Publisher’s note

All claims expressed in this article are solely those of the authors and do not necessarily represent those of their affiliated organizations, or those of the publisher, the editors and the reviewers. Any product that may be evaluated in this article, or claim that may be made by its manufacturer, is not guaranteed or endorsed by the publisher.
